# Small hole polaron in CdTe: Cd-vacancy revisited

**DOI:** 10.1038/srep14509

**Published:** 2015-09-28

**Authors:** A. Shepidchenko, B. Sanyal, M. Klintenberg, S. Mirbt

**Affiliations:** 1 Department of Physics and Astronomy, Uppsala University, Box 516, 75120 Uppsala, Sweden

## Abstract

The characteristics of electronic states of Cd-vacancies in CdTe, an important semiconductor for various technological applications, are under debate both from theoretical and experimental points of view. Experimentally, the Cd-vacancy in its negative charge state is found to have *C*_3*v*_ symmetry and a (−1/−2) transition level at 0.4 eV. Our first principles density functional calculations with hybrid functionals confirm for the first time these experimental findings. Additionally, we find that the *C*_3*v*_ symmetry and the position of the (−1/−2) transition level are caused by the formation of a hole polaron localised at an anionic site around the vacancy.

CdTe is an extensively used room temperature semiconductor detector material finding applications in a variety of fields, ranging from medical imaging to nuclear safeguards^1^,^2^. Because of a wide band gap, this detector does not require liquid nitrogen cooling in order to achieve acceptable resolution, cf. HPGe. Since the cooling systems for detectors are rather bulky, heavy and expensive, it gives a remarkable head start for those detectors, which can operate at room temperature, like CdTe and CdZnTe. Portable gamma-/X-ray semiconductor detectors and spectrometers are much-in-demand in today’s high-energy radiation industry, science and research. In addition, CdTe is the only II-VI semiconductor that easily can be doped both *n*-type and *p*-type^3^.

Cd(Zn)Te has been studied extensively both experimentally and theoretically. A lot of work has been done in order to achieve higher energy resolution, time efficiency and stability. It was established that imperfections of semiconductor crystals, such as native defects and impurities, which appear during crystal growth, are the main cause of insufficiently high technical characteristics of the detectors^4^. For example, in detection devices, where CdTe is a material of choice, any uncompensated defect gives rise to charge collection problems. During the decades of studies many defect and impurity energy levels in the band gaps of Cd(Zn)Te were experimentally determined and assigned, but not all of them were in agreement with theoretical models^5^. After about 50 years of studies this issue still remains unsolved.

One of the most important defects in CdTe is the Cd-vacancy. In particular, the negatively charged Cd-vacancy (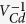) is assumed to be the dominant native *p*-type dopant^6^. Cd-vacancies are found both isolated and in defect complexes as A-centers^3^. Wienecke *et al.*^7^ concluded from high-temperature electrical measurements that for Te-rich CdTe, charged *V*_*Cd*_ are dominant. CdTe films subjected to thermal treatment in air become *p*-type^6^, because the film becomes Cd-deficient and Cd-vacancies are formed. Conductivity and Hall effect measurements were performed by Grill *et al.*^8^,^9^ on CdTe under both Cd-rich and Te-rich conditions. Based on a quasi-chemical defect model they conclude that the dominating defects are a Cadmium-interstitial as the divalent donor and a Cadmium-vacancy as the divalent acceptor. Photo induced transient spectroscopy (PICTS)^10^ on *p*-type CdTe reveals that the defect with the highest concentration is an electron trap located at 0.42–0.44 eV above the valence band maximum (VBM). It is existent for Ge-, Sn-, and Cl- doped CdTe. Thermoelectric-effect spectroscopy (TEES) and thermally stimulated current (TSC) experiments by Szeles *et al.*^11^ performed on Cd-vacancy enriched samples reveal a thermal ionisation energy of an acceptor level at 0.43 eV.

Electron paramagnetic resonance (EPR) experiments^12^,^13^ and electron nuclear double resonance (ENDOR) reveal the (−1/−2) transition level of the Cd-vacancy in CdTe to be less than *E*_*VBM*_ + 0.47 eV. Moreover, EPR experiments show that 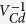 exhibits *C*_3*v*_ symmetry^12^ due to Jahn-Teller distortion. Yet, until now no Jahn-Teller distortion for the Cd-vacancy employing ab-initio calculations^5^,^14^,^15^,^16^,^17^,^18^,^19^,^20^ had been found. LDA calculations of the 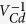 in CdTe reveal *T*_*d*_ symmetry of the ground state (Table 1) without any localized hole. Also, a metallic solution is found where the Fermi level lies within the defect level (see Fig. 1a) which is merged to the valence band edge both for nonmagnetic (not shown) and spin-constrained (Fig. 1a) LDA calculations. As there is no general consensus about the proper theoretical analysis of electronic and geometric structures of 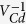 in CdTe, we have used methods going beyond LDA to clarify these issues. Moreover, we will show the emergence of a hole polaron due to the charged vacancy.

## Results and Discussion

### Ground state of 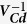


**Figure 1:**
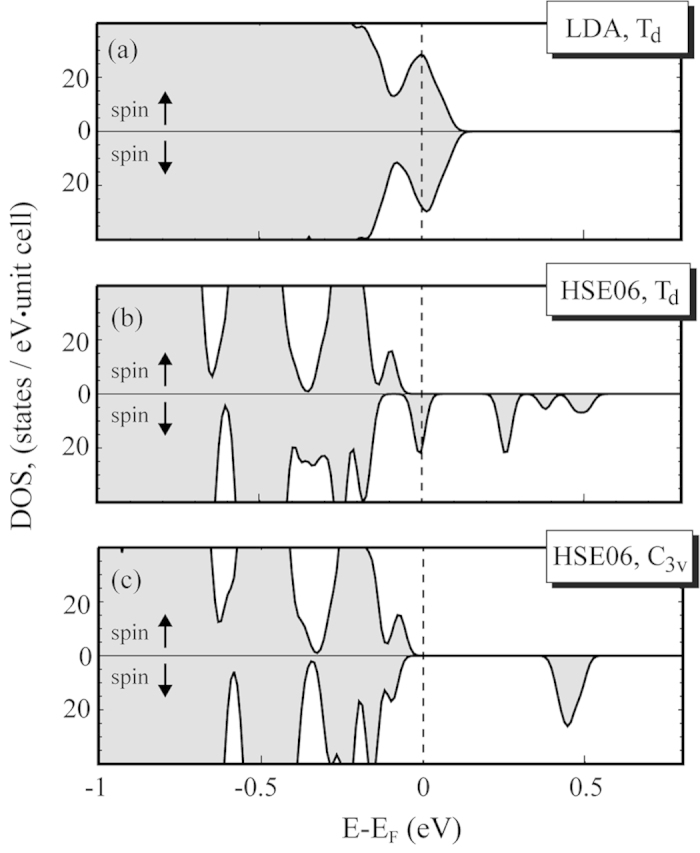
Spin-polarised densitiy of states of CdTe. In panels (**a**–**c**) results of the following calculations are shown: (**a**) LDA of 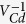 in *T*_*d*_ geometry; (**b**) HSE06 of 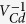 in *T*_*d*_ geometry; (**c**) HSE06 of 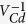 in *C*_3*v*_ geometry.

In our calculations, the spin-polarised HSE06 *C*_3*v*_ solution of the 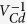 has the lowest energy. Relative to *C*_3*v*_, the nonmagnetic (spin-polarized) *T*_*d*_ solution is 42 meV (37 meV) higher. From the density of states shown in Fig. 1, we can conclude the following: the sum of the energy eigenvalues is lowest for the spin-polarized *C*_3*v*_ solution (Fig. 1c) because the peak at the Fermi energy has shifted to lower energies. All other solutions include a partially occupied peak at the Fermi energy. Indeed, the sum of the energy eigenvalues is calculated to be 190 meV lower for the *C*_3*v*_ geometry than for the *T*_*d*_ geometry.

Equilibrium lattice parameters, charge transition levels and local point group symmetries are tabulated in Table 1 showing values from previously reported literature along with our results obtained from HSE06 calculations. One can clearly see that our HSE06 calculations yield the (−1/−2) transition level in excellent agreement with the experimental value. Also, the point group symmetry of the ground state becomes *C*_3*v*_ in our HSE06 calculations, which is in agreement with experiments (Table 1).

In the isoelectronic ZnTe compound, Chan *et al.*^21^ studied the Zn vacancy and obtained a polaron solution by correcting the LDA potential using a hole-state potential operator. They calculated the *T*_*d*_ solution to be 90 meV higher than the *C*_3*v*_ solution and the (−1/−2) charge transfer level to be at 0.41 eV. The magnitude of our calculated Jahn-Teller distortion in CdTe is similar to the distortion in ZnTe.

### Geometry of 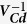


**Figure 2:**
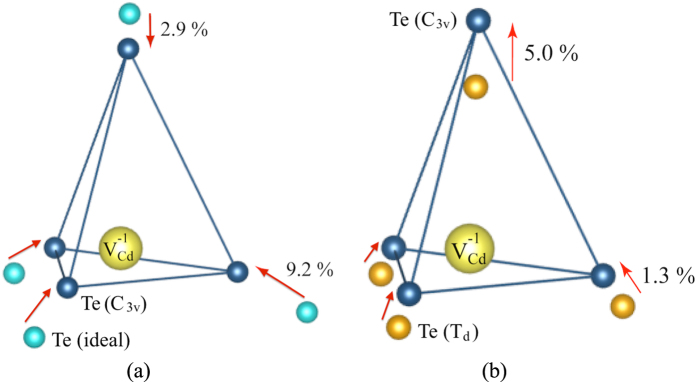
Geometry of the 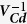. Relative shifts of the four Te-atoms (dark-blue circles) neighbouring the 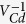 (yellow circle) with *C*_3*v*_ symmetry are shown with respect to the positions of the corresponding Te-atoms in (**a**) ideal CdTe (light-blue circles), (**b**) CdTe with the 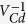 with *T*_*d*_ symmetry (orange circles). The distance between the Cd-vacancy and a Te-neighbor in ideal CdTe is taken as reference for the shift calculations given in %.

We compare the optimised geometries of the *T*_*d*_ and *C*_3*v*_ solutions of the 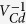 by analyzing the positions of the four nearest Te neighbors (Fig. 2). The light blue circles represent the positions of the Te-atoms in bulk CdTe. In accordance with *T*_*d*_ symmetry, they are placed at the vertices of a tetrahedron. The dark blue circles represent the Te positions around the 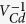 (yellow circle) after geometry relaxations with HSE06. In order to find the proper configuration of the hole polaron, we followed the Deskins-method^22^,^23^: The HSE06 calculations were done in two steps: First, one of the Te-atoms neighbouring the vacancy was replaced by Sb. The reason for this replacement is that Sb has one less electron than Te and thus provides a good approximation for the geometry of a Te-atom with a localised hole. Second, the Sb-atom was replaced with a Te-atom. With this recipe, the *T*_*d*_ symmetry of the initial configuration was broken and the 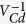 approached the *C*_3*v*_ solution with one localised hole. The 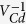 maintains the *C*_3*v*_ symmetry upon ionic relaxations. The resulting geometry was the following: three Te-atoms moved closer to the vacancy, each by 0.26 Å (9.2%) compared to ideal CdTe (Fig. 2a) and by 0.04 Å (1.3%) compared to CdTe with 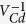 with *Td* symmetry (Fig. 2b); the Te-ion hosting the hole moved closer to the vacancy by 0.08 Å (2.9%) compared to ideal CdTe (Fig. 2a) and moved away from the vacancy by 0.14 Å (5.0%) compared to CdTe with 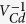 with *T*_*d*_ symmetry (Fig. 2b).

### Density of states of 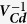


We compare the density of states of the *T*_*d*_ and *C*_3*v*_ solutions of the 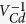 (Fig. 1). As we mentioned earlier, LDA calculations reveal a partially occupied defect level merged to the valence band edge (Fig. 1a). Nonmagnetic HSE06 calculations of the 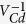 with *T*_*d*_ geometry still give a metallic solution (not shown). In our previous paper^24^ we showed that HSE06 calculations lowered the valence band edge by 0.26 eV. In addition, the defect state has now moved deeper into the band gap. The density of states of the spin-constrained calculation (magnetic moment equal to 1 *μ*_*B*_) of 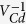 with *T*_*d*_ geometry is shown in Fig. 1b. This calculation only finds a solution with a *C*_3*v*_ magnetic symmetry in a *T*_*d*_ lattice geometry. The spin-up density of states is now completely occupied. On the other hand, for the spin-down channel we still find partial occupancy of the spin-down defect level closest to the valence band edge. The deep defect level from the nonmagnetic calculation is now split into four peaks, which most likely is a consequence of the mismatch between the magnetic and lattice symmetry. Finally, in Fig. 1c we show the density of states of a fully relaxed, spin-constrained HSE06 calculation with *C*_3*v*_ magnetic and lattice geometry. A semiconducting solution is found for 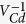. In addition, we find only one unoccupied defect level deep in the band gap in the spin-down channel.

### Existence of hole polaron

**Figure 3:**
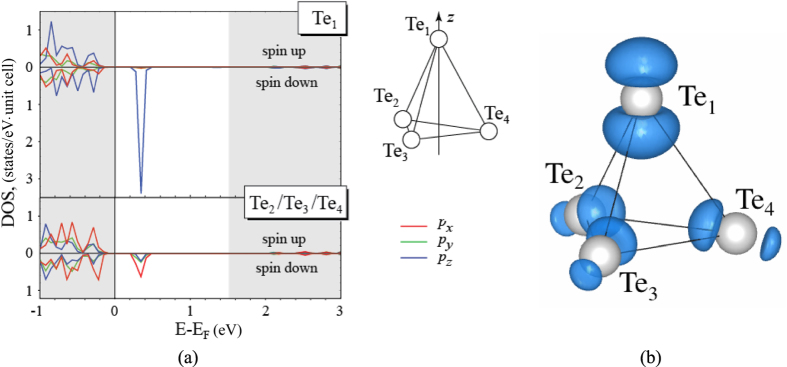
(**a**) Site and p-orbital projected density of states (HSE06) of the Cd-vacancy’s nearest neighbors (Te_1–4_) in the *C*_3*v*_ geometry. Upper panel shows results for Te_1_, the ion hosting the hole. Lower panel shows results for the remaining three equivalent Te neighbors. Valence and conduction bands are shown as shaded areas. (**b**) Isosurface (0.0017 electrons/Å^3^) of the partial charge density associated with the gap state visualizing the hole polaron.

Next, we discuss the localisation of the hole. In Fig. 3a, we show the site and p-orbital projected density of states of the vacancy’s nearest neighbours in the *C*_3*v*_ geometry. In the upper panel, we show the p-orbital density of states of the Te-neighbor (Te_1_) containing the hole. A strong p_*z*_-peak in the spin-down channel deep in the band gap is observed. The other three equivalent Te-neighbors (Te_2_, Te_3_, Te_4_) show a weaker contribution to the spin-down peak with mostly a p_*x*_ component. The remaining ions in our supercell do not contribute to the peak in the spin-down channel (not shown). The Te-Te distance between this three other Te-neighbors (Te_2_, Te_3_, Te_4_) is calculated to be 4.27 Å, which should be compared to the Te-Te dimer bond length of 2.78 Å.

In Fig. 3b, we show the partial charge density of the 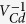 in the *C*_3*v*_ geometry. (We note that this charge density associated with the gap state looks identical to the magnetization density of the entire supercell.) It is seen that the largest charge contribution of the gap state is found on Te_1_, the Te-ion hosting the hole, with smaller but not negligible contributions on the other Te-neighbors. The remaining atoms in the supercell do not contribute to the hole polaron, which is why this hole polaron is termed a small polaron in accord with standard terminology^25^. We see that the hole polaron is asymmetric, where the density pointing towards the vacancy is larger than the density pointing away from the vacancy. The hole polaron has a p-like shape in agreement with the partial density of states shown in Fig. 3a. The analysis of the partial charge density confirms and depicts the existence of the small hole polaron accompanying the negatively charged Cd-vacancy in CdTe.

The hole polaron formation accompanying the 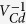 in CdTe was never described before by means of ab-initio methods. Some speculations have been made^26^,^27^ regarding a Jahn-Teller distortion of the 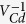 in CdTe in analogy to the Zn vacancy in ZnSe. In fact, a general model for the localised hole plus trigonal distortion has been proposed for BeO, ZnO, ZnSe, ZnS, and CdS^26^. In this paper, we show explicitly that in CdTe, the breaking of the tetrahedral symmetry is achieved by a trigonal Jahn-Teller distortion, where the symmetry changes from *T*_*d*_ to *C*_3*v*_, which is in agreement with the experimental hyperfine structure^12^,^13^. The localized hole associated with this symmetry lowering is highly p-like and points towards the vacancy as seen in Fig. 3.

### Summary

In summary, employing HSE06 calculations, we find for the first time the ground state of the 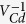 in CdTe to have *C*_3*v*_ symmetry in agreement with EPR experiments. Our calculated (−1/−2) transition level agrees excellently with experiments in contrast to all previous LDA calculations. In addition, our analysis shows that the ground state of the 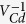 is stabilised by the formation of a small hole polaron breaking the tetrahedral symmetry around the Cd vacancy. These results indicate the importance of going beyond LDA for studying defects in semiconductors.

## Methods

Our calculations were performed using the VASP^28^ (Vienna Ab-initio simulation package) code. Two types of calculations were performed: first, based on density functional theory (DFT) within the local density approximation (LDA)^29^,^30^ parametrized by Ceperley and Alder (CA)^31^; and second, the screened hybrid functional of Heyd, Scuseria and Ernzerhof (HSE06)^32^,^33^ employing a screening parameter of 0.2 Å^−1^ and mixing parameter of 0.25. The suitability of using HSE06 in describing correctly polaronic features in defected semiconductors has already been established^22^. The electron-ion interactions were described by projector augmented wave (PAW) pseudopotentials. Configurations for valence electrons were −4d^10^, 5s^2^ and 5s^2^, 5p^4^ for Cd and Te respectively.

Comparing the experimental bulk modulus of CdTe, (45 GPa), with the calculated (HSE06) bulk modulus (40 GPa), we conclude that our HSE06 calculation describes the elastic properties of CdTe rather well. With HSE06 (LDA) the lattice constant is 1.5% (1.1%) too large (small) compared with experimental lattice constant^34^ of 6.48 Å. The band gap is calculated with HSE06 to be 1.53 eV which is in good agreement with experiment^34^ (1.56 eV). (LDA instead gives a value of 0.64 eV.)

The valence wave functions were expanded in a plane-wave basis with a cut-off energy of 350 eV. Our calculational unit cell had 128 atoms. For geometry optimizations, the atoms were relaxed until the Feynman-Hellman forces on each atom reached 0.01 eV/Å. For the k-point sampling we used a Monkhorst-Pack (for LDA) and Gamma-centered (for HSE06) (2 × 2 × 2) grid with a Gaussian smearing of 0.025 eV, which was earlier shown to reproduce converged features of the CdTe valence band^24^. As discussed earlier^24^, the (2 × 2 × 2) k-point grid is not sufficient to obtain a smooth conduction band edge but fortunately both the Cd vacancy and Te antisite defect states originate from the valence band and we concluded that the insufficient resolution of the conduction band edge is negligible in the context of the here presented results. Cd-vacancies (*V*_*Cd*_) were simulated by taking away one of the Cd-atoms in the CdTe supercell. For the 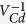 and 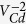, one and two electrons were added to the total number of electrons for the neutral system. Charged defects were calculated with a charge-compensating background to ensure convergence of the total energy. For 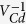, spin-polarised calculations were performed and, taking into account the semiconductor nature of the material modelled, the spin moment was constrained to 1 *μ*_*B*_.

The formation energies Δ*H*_*f*_ (*E*_*F*_) for Te-rich conditions were calculated as follows:


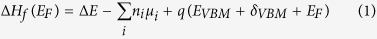


where Δ*E* is the energy difference between the total energy of the defect supercell in a charge state *q* and the corresponding total energy of the neutral defect free system. *n*_*i*_ is the difference in the number of atoms for the i-th atomic species between the defect-containing and defect-free supercells. *μ*_*i*_ is the chemical potential of the i-th atomic species, *E*_*VBM*_ is valence band maximum of the bulk CdTe. *E*_*F*_ is the Fermi level, i.e. the electron chemical potential regulating if electrons are available to charge the defect. Total energy calculations of charged defects in finite-size supercells include unwanted defect-defect interactions^35^,^36^. The defect concentration in our supercell is on the order of 10^20^ cm^−3^, whereas typical native defect concentrations are only on the order of 10^15^ cm^−3^. For charged defects, the interaction between periodic images should be corrected in an appropriate manner. Many of the known correction schemes are either computationally too expensive for HSE06 calculations or not generally reliable to minimize the errors^35^,^36^. On the other hand, a computationally cheap and reliable correction scheme is “potential realignment^37^,^38^,^39^”, where the potential in the defect cell is aligned to that of bulk by *δ*_*VBM*_. For *δ*_*VBM*_ we use the difference between the potential of the most unperturbed point in the defect-containing supercell and the same point in the defect-free supercell^40^. We calculate for 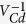 (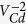) *δ*_*VBM*_ = −0.04 eV (*δ*_*VBM*_ = −0.17 eV)

## Additional Information

**How to cite this article**: Shepidchenko, A. *et al.* Small hole polaron in CdTe: Cd-vacancy revisited. *Sci. Rep.* **5**, 14509; doi: 10.1038/srep14509 (2015).

## Figures and Tables

**Table 1:** Calculated (0/−1) and (−1/−2) transition levels of *V*_*Cd*_ in CdTe in eV relative to VBM.

Method	0/−1)	(−1/−2)	*Q*_*corr*_	*a*_0_ [Å]	*N*	*P*	ref.
LDA	0.10	0.36	MP	6.44	216	*T*_*d*_	15
LDA	0.18	0.26	PR	6.42	64	*T*_*d*_	16
LDA	0.13	0.21	—	6.54	32	*T*_*d*_	17
LDA	0.12	0.27	—	6.48	64	*T*_*d*_	18
GGA	0.10	0.25	MP	6.62	64	*T*_*d*_	19
LDA	−0.01	0.04	no	6.45	216	*T*_*d*_	15
LDA	−0.05	0.06	PR	6.41	128	*T*_*d*_	
HSE06	0.38	0.44	PR	6.58	128	*C*_3*v*_	
Exp.		0.43–0.47		6.48		*C*_3*v*_	10, 11, 12

*Q*_*corr*_ is the employed charge correction method, where MP and PR denote Makov-Payne correction^39^ and potential realignment method^37^ respectively. *a*_0_ is the employed lattice constant of CdTe. *N* is the number of atoms used in the supercell. *P* indicates the point group of the calculated ground state of 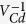.
